# Antifungal Metabolites as Food Bio-Preservative: Innovation, Outlook, and Challenges

**DOI:** 10.3390/metabo12010012

**Published:** 2021-12-23

**Authors:** Bishwambhar Mishra, Awdhesh Kumar Mishra, Sanjay Kumar, Sanjeeb Kumar Mandal, Lakshmayya NSV, Vijay Kumar, Kwang-Hyun Baek, Yugal Kishore Mohanta

**Affiliations:** 1 Department of Biotechnology, Chaitanya Bharathi Institute of Technology, Hyderabad 500075, India; mishra.bishwambhar@gmail.com (B.M.); sanjeeb.vit@gmail.com (S.K.M.); lakshmannunna@gmail.com (L.N.); 2 Department of Biotechnology, Yeungnam University, Gyeongsan 38541, Gyeongsangbuk-do, Korea; awdhesh@ynu.ac.kr (A.K.M.); vijaykumarcbt@gmail.com (V.K.); 3 Department of Biotechnology, National Institute of Technology, Tadepalligudem, Andhra Pradesh 534101, India; sanjay0193@gmail.com; 4 Department of Orthopedics Surgery, Johns Hopkins University School of Medicine, Baltimore, MD 21205, USA; 5 Department of Applied Biology, University of Science and Technology Meghalaya, Ri-Bhoi 793101, India

**Keywords:** anti-fungal, bio-preservation, food spoilage, perishable foods, shelf life

## Abstract

Perishable food spoilage caused by fungi is a major cause of discomfort for food producers. Food sensory abnormalities range from aesthetic degeneration to significant aroma, color, or consistency alterations due to this spoilage. Bio-preservation is the use of natural or controlled bacteria or antimicrobials to enhance the quality and safety of food. It has the ability to harmonize and rationalize the required safety requirements with conventional preservation methods and food production safety and quality demands. Even though synthetic preservatives could fix such issues, there is indeed a significant social need for “clean label” foods. As a result, consumers are now seeking foods that are healthier, less processed, and safer. The implementation of antifungal compounds has gotten a lot of attention in recent decades. As a result, the identification and characterization of such antifungal agents has made promising advances. The present state of information on antifungal molecules, their modes of activity, connections with specific target fungi varieties, and uses in food production systems are summarized in this review.

## 1. Introduction

The world’s population is expected to reach 9.7 billion people by 2050 [1]. With the growing population, food waste and deterioration must be significantly reduced. As a result, the food business is confronting significant hurdles in meeting present and future demand. Aside from challenges such as food warehousing and distribution infrastructure, climate change impacts, and water resilience, there is far too much food waste, which encompasses livestock and crop illness.

To some extent, the food industry provides a solution through the use of admixtures such as artificial preservatives, which allow manufacturers to meet customer demands for diverse array, ease of access, price, convenience, and delivery performance while reducing the amplitude of technological treatments that results in quality losses [2]. On the other hand, consumers are not atypical in their condemnation of some food additives. Moreover, awareness among consumers about food safety and hygiene and their rejection of chemical additives has prompted research into the use of beneficial microorganisms and their metabolites as viable natural preservatives for storage stability and improved food safety.

Managing foodborne pathogens in a wide range of food items is a significant concern for the food business, which could be solved by strategically using bio-compounds or bio-preservatives. Fungi can potentially pose important challenges during the processing of food. Deteriorative fungus plays a key role in the deterioration of perishables such as sauces, sweetened beverages, and cheese. Fungi are a common cause of food decomposition. The bulk of fungal species are saprobic, meaning they have adapted to obtain their nutrients from dead organic waste. These fungi are chemoheterotrophic, meaning they each have a set of extracellular enzymes capable of dissolving structured biopolymers during vegetative decomposition in a complex ecosystem. In food, fungi follow a similar pattern. Canonical fungal ecology, on the other hand, frequently assesses decomposition and nutrient status in complex polycultures, whilst in food processed for shelf life extension and stability, the microbial population is greatly eroded, which, when blended with commodity use performance, increases the likelihood of contamination reliant on a single main competitor [3].

Recognition of precise spoilage fungi has stepped forward substantially since the 1990s because of the arrival of molecular strategies and global taxonomic consensus. For example, identifying a restrained subset of fungi responsible for food spoilage in a particular product permits the improvement of centered prevention and intervention techniques that lessen food waste [4]. Toxicants of microbial origin, as well as disease-causing pathogens such as *Salmonella*, *Staphylococcus aureus*, *Escherichia coli*, *Bacillus cereus*, *Aspergillus niger*, and *Clostridium perfringens*, might very well pose a serious health risk to consumers [5], with admixtures posing an even greater risk [5]. Consequently, adopting naturally occurring substances not only prevents and limits the spread of undesirable bacteria but it will also enhance food quality and safety and reaffirm buyers’ faith in the trustworthiness of their food [6].

Bio-preservatives are naturally occurring substances derived from plants, animals, and microbes that prolong the shelf life of food [7]. These substances suppress pathogenic organisms in food to a bare minimum or even eradicate them entirely while also improving food function and quality. Many of these chemicals are antimicrobials as well as antioxidants, and they tear down cell membranes and disrupt biosynthetic bacteria pathways [8]. The major elements of antifungal metabolites and their methods of action, interactions with their target fungi types, and their uses in food systems are highlighted in this concentrated study. The extent of prospective research directions, as well as main hurdles, are also summarized.

## 2. Perishable Food Ecosystem and Microbiota

Perishables are foods that decay, deteriorate, or become unhealthy to eat if not properly stored or devoured promptly after purchase. These foods include meat, seafood, dairy products such as milk and cheese, poultry, as well as fruits and vegetables. Once the food’s usual state alters adversely, it is said to have deteriorated [9]. It could be a difference in the scent, taste, mouthfeel, or appearance of the food. The most prominent causes of rotting food are bacteria, molds, and yeasts.

### 2.1. Fungal Spoiler of Foods

The heterogeneity of fungus and yeast as culinary spoilers has been studied in many studies and food commodities. For example, fungal species, such as *Saccharomyces* *cerevisiae* in fermented products, *Penicillium camemberti* in mold-ripened cheeses, and *Aspergillus oryzae* in soy sauce, are not necessarily harmful to food production, and some are even required to provide the appropriate organoleptic features [10]. However, it is also worth remembering that a common fungus species found in fermented products can turn out to be a narrative surprise in another product.

Due to changes in pH, carbohydrates, surface, and resistance that create ideal conditions for fungal spoilers, fruits and vegetables are particularly sensitive to mold growth throughout the maturation phase. For example, fungi cause observable signs on postharvest crops, such as discoloration and tissue abscess formation. Citrus, berry, stone, pome, tropical, and solanaceous fruits all become spoiled due to their presence. In addition, fruit and vegetable fungal diseases have been extensively studied [11]. Rhizomes and other vegetables such as crucifers, cucurbits, bulbs, and legumes are less susceptible to fungal illnesses than fruits, possibly because their pH is more favorable to bacterial pathogens [12]. In addition, many of the most common postharvest illnesses are caused by fungal species from the *Penicillium*, *Monilinia*, *Botrytis*, *Alternaria*, *Rhizopus*, *Fusarium*, *Aspergillus*, *Gloeosporium*, *Geotrichum*, and *Mucor* genera [13].

Fish and milk are less prone to fungal deterioration than other animal-derived meals and raw materials, but bacterial spoilage is significantly affected. Even if the bacterial deterioration is predominated, fungal spoilage can develop in meat, especially during refrigeration [14]. Notwithstanding the need for more excellent research, black patches have been found in *Penicillium*, *Clostridium*, and *Aureobasidium* species, while *Thamnidium* spp. can generate ‘whiskers’ on carcasses. Yeast from the *Candida*, *Cryptococcus*, and *Yarrowia* species have been found on moist, packed foods, causing off-flavors and cosmetic flaws, including slime and stains [15].

### 2.2. Antifungal Microorganisms in Foods

As already mentioned, bio-preservation is gaining traction as a technique of improving food quality and safety in response to the widespread desire for less refined foods free from preservatives [16]. In recent years, many antifungal strains from a variety of microbial species have been found. They have been detected in vegetables, fruits, meat, cereals, milk and other food-related products. In addition, researchers have recently extended the isolation of new bioprotective cultures to different settings, including deep-sea and Arctic soil samples, so as to identify microorganisms potentially releasing new antifungal chemicals [17].

**Figure 1:**
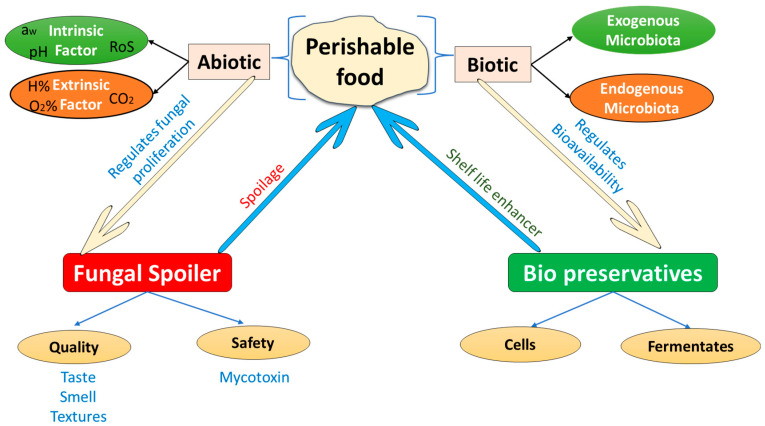
Schematic representation of perishable food ecosystem and their interaction between food spoilers and bio-preservatives.

Antifungal activity levels and the spectrum of inhibited fungal targets differ greatly depending on the examined species and strain to strain within a species, necessitating the use of screening procedures to identify efficient antifungal microorganisms (Figure 1). For example, there was up to 75% difference across five *Lactobacillus casei* strains assessed for their ability to suppress the growth of four spoilage molds [18]. In another study, just a few *Lactobacillus plantarum* isolates out of 88 investigated showed a wide range of fungal inhibition [19]. In another investigation, only a few *L. plantarum* isolates out of 88 examined demonstrated a broad spectrum of fungus inhibition [20]. Another study found that 55 yeast isolates from the skin of grape varieties (*Aureobasidium pullulans*, *Cryptococcus magnus*, *Candida zeylanoides*, *Pseudozyma aphidis*, *Candida sake*, *Hanseniaspora* *uvarum*, *Rhodotorula mucilaginosa*) could hinder *Aspergillus tubingensis* cell growth, with a 58% inhibition.

## 3. Classification of Antifungal Metabolites Found in Food Habitats

Metabolites that can control the fungal infection or disease are categorized as antifungal metabolites. These metabolites are produced and accumulated by a wide range of bacterial and fungal species, actinomycetes, and plants. Several genera of lactic acid bacteria (LAB) have been reported to produce various antifungal compounds, viz. phenyllactic acids, fatty acids, organic acids, reuterin, cyclic dipeptides, diacetyl, hydrogen peroxide, lactones, and alcohols [21]. There are various groups of antifungal metabolites found in food habitats, as follows.

### 3.1. Organic Acids

Organic acids have been utilized for several years to prevent microbial growth in food products such as bread and sausages [22,23], milk and milk-based products [24], meat and poultry products [25], and fruits and vegetables [26,27]. A few examples of major organic acids used as antifungal agents which target various fungal species are described in Table 1.

### 3.2. Phenyllactic Acid (PLA)

Phenyllactic acid is a natural bioactive compound, having a broad-spectrum inhibitory activity against a few bacteria (*Enterococcus* spp., *Listeria monocytes*, *Salmonella* spp., *Staphylococcus aureus*, etc.), fungi, molds, and yeast (*Candida* and *Rhodotorula* spp.) [36]. Typically, PLA is obtained from LAB such as *Lactobacillus*, *Weissella*, and *Leuconostoc* through phenylalanine metabolism. In this process, aromatic aminotransferase enzyme transaminates phenylalanine to phenylpyruvic acid (PPA), which is further reduced to PLA by lactate dehydrogenase enzyme [37,38]. LAB inhibits undesirable microbial and fungal growth by reducing the pH level in the system. Hence, PLA produced by LAB improves food safety with increased shelf life of food products, which subsequently positively impacts consumers’ health. Therefore, PLA has been widely accepted as a natural preservative in the food industry [39,40]. A few examples related to the sources of PLA and their antifungal activity against a broad spectrum of microbes along with the associated food products are described in Table 2.

### 3.3. Fatty Acids

Fatty acids are accumulated by a broad spectrum of microbes (such as bacteria, actinomycetes, and fungi) as well as plants. Various literature has revealed that fatty acids have been used as an active antimicrobial agent against a wide range of food spoilage bacteria, molds, and yeast [58,59,60]. A study [61] highlighted the correlations between the configurations of hydroxy unsaturated fatty acids (HUFA) and their associated antifungal activities. This study also exhibited that the 18-carbon unsaturated fatty acid chains having the hydroxyl group in the center’s proximity showed intense antifungal activity [61]. Similarly, Souza et al. [62] explained the antifungal activity against *Candida* species concerning change in the structure of aliphatic fatty acids. They also described that the structure of each fatty acid strongly affects its antifungal activity. The antifungal activity is strongly affected by the carbon chain length; for example, an increase in carbon chain length reduces the antifungal activity of fatty acids (and vice versa), whereas medium chain length fatty acids usually reveal the maximum antifungal activity [62]. Furthermore, the hydroxyl group was found to be essential for antifungal activity. It was also reported that the capric and lauric acids had shown the best anti-*Candida* results.

Elsherbiny et al. [63] studied the anti-*Penicillium* effect of β-aminobutyric acid (BABA) in orange fruit. They reported that the concentration of BABA plays a crucial role in the inhibition of fungal growth; for example, growth of fungal strain was significantly inhibited by 125 mM of BABA. Pinilla et al. [64] examined the antifungal property of oleic acid through liposomes of oleic acid (OA) and phosphatidylcholine (PC) encapsulating garlic extract (GE). It showed great potential for controlling fungal growth in wheat bread. *Lactobacillus* sp. RM accumulate 6-octadecenoic acid and hexadecanoic acid as a secondary metabolite, which is reported as an effective antifungal agent against mycelia of *Aspergillus parasiticus* [65]. 3-Hydroxy-5-dodecenoic acid causes severe damage to the surface of *Bacillus cereus* and leads to a decrease in the endospore size of the cell. Solano et al. [58] reported that the intermediate concentration of lauric acid exhibited strong antifungal activity against *Colletotrichum tamarilloi.*

### 3.4. Reuterin

Reuterin (3-hydroxy propionaldehyde or 3-HPA) is a non-proteinaceous and water-soluble antimicrobial agent produced by *Lactobacillus reuteri* [66]. Other genera of bacteria have produced it, including *Citrobacter*, *Bacillus*, *Klebsiella*, *Enterobacter*, and *Clostridium* [67]. Reuterin was reported to be produced during the anaerobic metabolism of glycerol (Vollenweider and Lacroix, 2004). Reuterin can work over a broad range of pH and remain active in the presence of various enzymes [68]. Therefore, reuterin was reported as a potential antibacterial and antifungal agent against a broad spectrum of microbes and utilized as a food preservative in the food industry [69]. Several inhibitory activities of reuterin have been investigated against a broad spectrum of microbes. However, the studies related to the antifungal activity of reuterin are limited as the inhibiting lead molecules present in reuterin have not yet been investigated so far.

A study on minimum fungicidal activity and inhibitory activity of reuterin against a group of fungi and yeast species reported that the reuterin produced by *Lactobacillus reuteri* ATCC 53608 showed the inhibition of growth of food spoilage microorganisms at a concentration of 11 mM or less. Additionally, reuterin also exhibited a fungicidal activity (99.9%) at concentrations equal to or below 15.6 mM [67]. At the same time, another study reported that the reuterin was an effective antimicrobial agent against *Lactobacillus delbrueckii* sp. *Bulgaricus*, *Penicillium expansum*, *Listeria monocytogenes*, *Staphylococcus aureus*, and *Escherichia coli* DH5α microorganisms. There are few microorganisms that are highly resistant (*Lactobacillus reuteri* ATCC 53608), and few are susceptible (*E. coli* DH5α) to reuterin [69].

### 3.5. Cyclic Dipeptides (CDP)

Cyclic dipeptides are the secondary metabolites that have been isolated from several species of bacteria [70], such as *Bacillus cereus* subsp. *thuringiensis* [71], *Lactobacillus plantarum* [72], and *Bacillus velezensis* AR1 [73]. CDPs are active at higher temperatures and resistant to denaturation by hydrolytic enzymes [74]. Individual CDPs worked as bio-effectors, whereas pooled CDPs have shown potential bioactive properties against numerous pathogens. In addition to that, the combination of CDPs in the presence or absence of antibiotics may exert collaborative antimicrobial properties [75]. As an antimicrobial function, CDPs may decrease the mycelial growth, alter the nuclear DNA functionally and structurally, make the mold membrane porous, inhibit ergosterol synthesis, alter the osmotic equilibrium, and initiate the oxidative apoptotic stress [76]. Thus far, various studies have been conducted to identify the newer CDPs as potential antifungal agents and antibacterial agents. Recent investigations on CDPs as having potential antifungal agents are described in Table 3.

### 3.6. Miscellaneous Antifungal Compounds

Although organic acids, phenyllactic acid, fatty acids, reuterin, and cyclic dipeptides are the major antifungal compounds synthesized and accumulated by a range of microbial strains associated with food habitat, a few other compounds of microbial origin (such as nisin, lactocin, divergicin and hydrogen peroxide) were also reported as potential antifungal agents [87]. In addition to that, some other antifungal compounds are also being reported to be accumulated as well as plants. Caffeic acid and rosmarinic acid extracted from *Lamiaceae* herbs showed strong antifungal activity against the *Fusarium oxysporum* f. sp. *Cyclaminis* [88]. Essential oils are volatile substances and are naturally produced by plants as secondary metabolites. These are known for their antifungal, insecticidal, and antibacterial properties. The essential oils extracted from spices (garlic and clove) showed antimicrobial activity against fungal pathogens such as *Candida albicans*, *C. catenulate*, *C. acutus*, *C. apicola*, *C. tropicalis*, *C. inconspicua*, *Trigonopsis variabilis*, *Rhodotorula rubra*, and *Saccharomyces cerevisiae* [89].

## 4. Mode of Action for Various Metabolites

As mentioned in the previous section, we have many diversified categories of antifungal metabolites ranging from organic acids to modified PLA, along with several substances such as reuterin, cyclic dipeptides, etc. Though they prove their efficacy and efficiency in various ways, they might involve chemical treatment at some of the other stages. Although they have limited side effects, they could be significant in the long run and with prolonged usage. Although biosynthesis proves to be a promising alternative for these chemical substances, this approach may not exactly resemble the chemical counterpart of the same. However, we can overcome this barrier by looking onto natural derivatives rather than a natural way of synthesizing the chemical components. This section highlights several aspects of biosynthesis, derivation as well as mechanisms [90,91].

### 4.1. Citric Acid and Phenyllactic Acid

**Figure 2:**
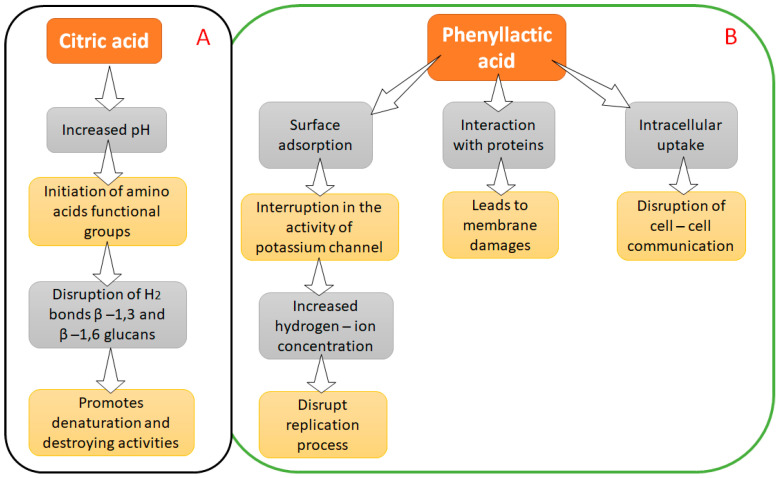
Mechanism of organic acids ((**A**) for Citric acid; (**B**) Phenyllactic acid) as bio-preservatives.

Among the many organic acids (which are generally weak acids), citric acid shows as high as 80% antifungal properties. Citric acid can be biosynthesized using fungal fermentation, either liquid surface fermentation or submerged fermentation. The ability of citric acid to inhibit mycelial growth proves its efficacy as an antifungal agent (Figure 2) [92]. Because of their solubility, flavor-enhancing qualities, and low toxicity, organic acids are commonly utilized as antibacterial or acidulant preservatives in the food industry. Sorbic acid and its sodium, potassium, and calcium salts are widely used as powerful antifungal and antibacterial agents, extending the shelf life of food goods.

Organic acids producing bacteria comprise the larger class of LAB (lactic acid bacteria), which have been used in the food industry for a long time. Organic acids are extensively synthesized from lactic acid bacterial species such as, *Pediococcus acidilactici* which can be cultivated in labs or even found in traditional Chinese medicines [93]. The whole class of LAB shows a wide range of mechanisms depending upon the species used as an antifungal agent. This broad spectrum includes increased oxidative stress and cell permeability, enzyme inhibition, proton gradient interference, etc. [94,95]. Phenyllactic acids (PLA) (also called 3-Phenyl lactic acid or 2-Hydroxy-3-Phenylpropionic acid) inhibited *Penicillium roqueforti*, *Aspergillus ochraceus*, *Fusarium graminearum*, *Penicillium expansum*, *Aspergillus niger*, *Monilia sitophila*, *Aspergillus flavus*, *Penicillium verrucosum*, *Penicillium citrinum*, and other fungi [96]. In a study, PLA had a minimum inhibitory concentration (MIC) of 6.5–12.0 mg/mL against fungus [20]. The mechanism of the antifungal activity of PLA is poorly understood. Various researchers have suggested that PLA interferes with the proton gradient and inhibits cellular enzymes, often coactively working with other metabolites [97]. PLA’s antifungal activities are thought to be inhibiting fungal radial growth and sporulation. PLA also inhibited the development and sporulation of fungal radicals on malt extract agar [98].

### 4.2. Essential Oils and Phytochemicals

**Figure 3:**
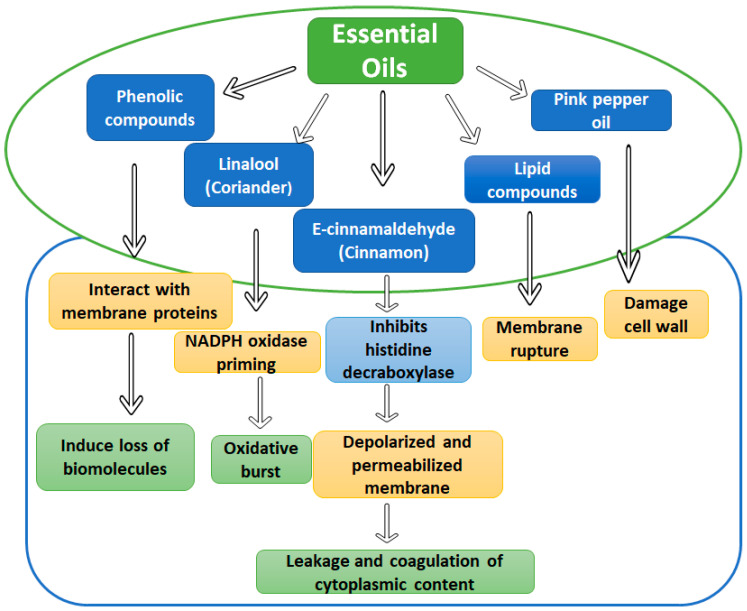
Mechanism of various essential oils as bio-preservatives.

Essential oils are the substances released by plants as a defense mechanism against extraneous factors. They can be easily extracted from various parts of plants such as flowers, stems, roots, leaves, etc. They have also been used as perfumery agents for centuries. Though the number of EOs produced by plants is relatively high, it would be a sophisticated process to characterize every EO, synthesis, and mechanism. Therefore, a few of them have been summarized in Figure 3 below [99].

**Figure 4:**
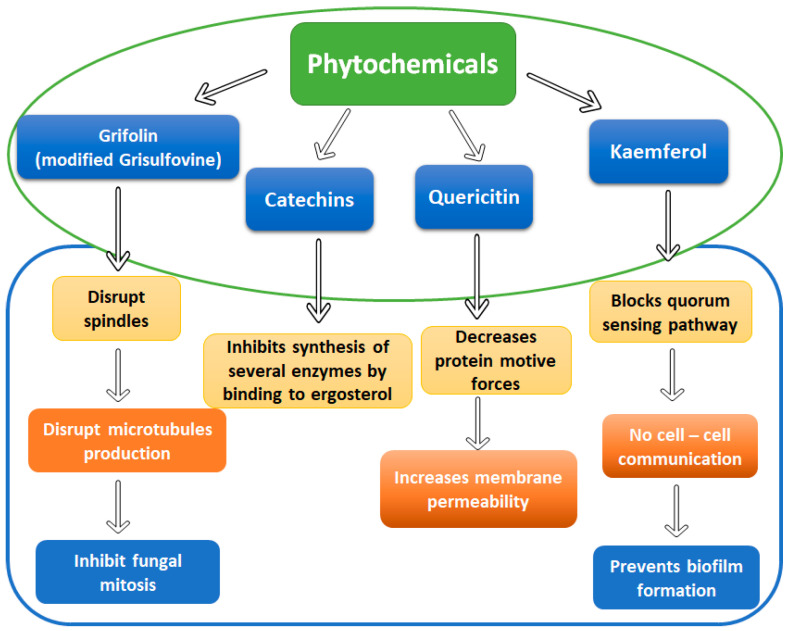
Mechanism of various phytochemicals as bio-preservatives.

Terpenes are the most diverse category of chemical compounds identified in plant extracts, with significant antifungal action that can be boosted synergistically by the presence of additional phytochemicals (Figure 4). Grifolin, a sesquiterpene chemical derived from the fruiting bodies of the fungus *Albatrellus dispansus*, inhibits the mycelial growth of plant pathogenic fungi such as *Sclerotinina sclerotiorum*, as well as spore germination on *Fusarium graminearum*, *Pyricularia oryzae* and *Gloeosporium fructigenum* [100]. Catechins were shown to rupture the fungal membrane by binding to the ergosterol layer and inhibiting the intracellular and extracellular enzymes [101]. On the other hand, Quercetin proves its antifungal activity by decreasing protein motive forces, thereby increasing membrane permeability [102]. Kaemferol works by blocking the QS pathway, which leads to failure of the cell-to-cell communication which ultimately prevents biofilm formation [103].

### 4.3. Azoles

**Figure 5:**
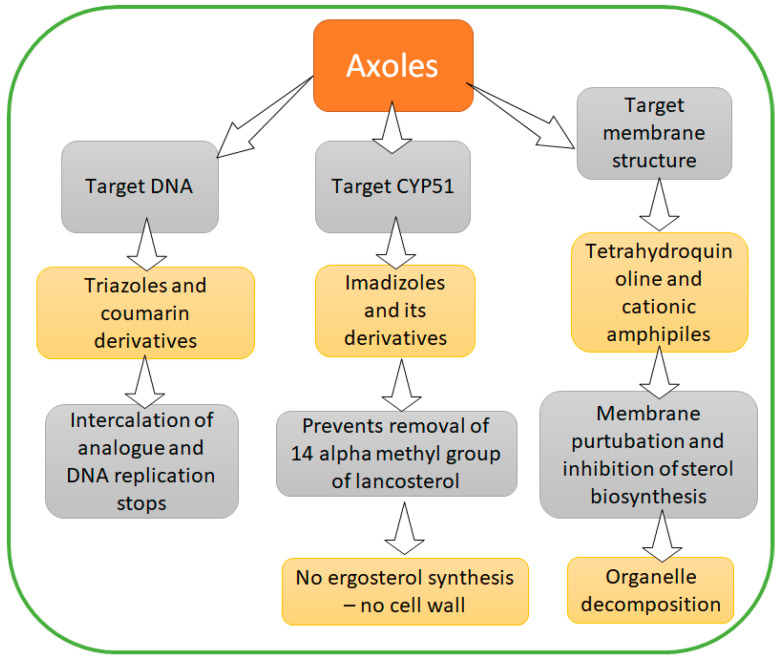
Mechanism of azoles as bio-preservatives.

Azoles are another class of excellent antifungal agents, which target the fungal cell membrane by acting as competitive inhibitors for CYP51 (a cytochrome P450 enzyme). CYP51 plays a vital role in ergosterol biosynthesis (which is the main component of the fungal cell wall). In addition, the class of azoles includes various sub-components acting as potential antifungal agents that can be categorized based upon their targeting molecules (Figure 5) [104].

## 5. Applications Oriented Studies from Laboratory to Pilot Scale

Conventional suspensions prepared from phytocompounds have antifungal effects. The antifungal range of a nanoemulsion made by ultrasonication using *Cleome viscosa* essential oil and Triton-x-100 was studied. Essential oil nanoemulsion (EONE) was evaluated with foodborne pathogenic *Candida albicans* at a minimum inhibitory and fungicidal dosage. The MIC and MFC values for *C. albicans* isolates ranged from 16.5 to 33 mL/mL, with a considerable reduction in biofilm. Fourier transformed infrared spectroscopy corroborated the shift in compositional fingerprinting, and spectroscopic analysis revealed a drop in chitin levels in cell walls. In *C. albicans* cells, EONE and its biologically active compounds cause massive damage [105].

Several techniques have proven that the primary components of EOs have antioxidant, antibacterial, and antifungal effects. Tea tree oil, lemon oil, cinnamon oil, clove oil, and thyme oil, among other EOs from local plants, have positively influenced antibacterial and antioxidant activity, along with expanded cereal shelf lives and enhanced food security. In addition, terpenes and volatile aromatic chemicals, for example, are important EO categories that help food hygiene without affecting quality. For example, EOs might be utilized as an additional preservative to extend the shelf life of grains and cereals because of their numerous effects, including antioxidant and antibacterial properties [106].

The antifungal and anti-aflatoxigenic activities of 5′-hydroxy-aurapten (5′-HA) on *A. flavus* isolated from nuts (*Lotus lalambensis*) were investigated. In this study, 5′-HA demonstrated a higher antifungal potential against *A. flavus,* having a minimum inhibitory concentration of 62.5 mg/L. It was found that 5′-HA had reduced conidia germination for *A. flavus* by 60% at a dose of 40 mg/mL in the early (A, B, C), middle (L, M, N), and late (P, Q, W) stages of the aflatoxin biosynthesis pathway. Moreover, 5′-HA also inhibited the synthesis of aflatoxins, AFB1 and AFB2, by 50% and 23.3%, respectively. 5′-HA increased the efficacy of enzymatic antioxidants CAT (Catalase) and SOD (Superoxide dismutase) by 56.25% and 66.66%, respectively. The anti-aflatoxigenic mechanism of 5′-HA is thought to work by increasing the expression profile of the transcription factors *atfA* and *atfB* by 2- and 2.5-fold, respectively [107]. Sodium lignosulfonate was found to be an antifungal compound due to its fungistatic activity against *M. circinelloides*, *A. amoenus*, and *P. solitum.* These strains were obtained from spoiled alfalfa hay (*Medicago sativa*). Sodium lignosulfonate (NaL) had superior preservation properties for the ground high-moisture hay as a substrate [108]. In comparison to spoiled hay, sodium lignosulfonate and PRP had a protective effect against hay proteolysis at a concentration of 0.5%, as assessed by a decrease in ammoniacal nitrogen (NH_3_-N). Preservatives can prevent plant proteins from deteriorating, retaining their biological worth, according to these studies.

Natamycin is an antifungal medicine with poor solubility that is used in food products to address the base of cheese and sausages. This use does not risk the customer’s safety. For beverage preservation, a highly soluble natamycin–cyclodextrin integral membrane was created. This approach results in high drug concentrations that are dangerously above the acceptable limit. In addition to assessing an adequate daily natamycin food intake, researchers must investigate natamycin’s impact on the intestinal bacteria as a reservoir for tolerance, which results from the amount of feces in one’s system to be abnormally high. Foods having natamycin, introduced and blended uniformly, such as yoghurt, and even the administration of cyclodextrin intercalation to drinks and wine, all contribute to natamycin levels and fecal *Candida* spp. drug exposure. *Candida* spp. have established natamycin tolerance in the bowels of persons who have been treated with natamycin for fungal diseases. As a consequence, it is impossible to figure out the likelihood that using natamycin to keep yoghurt and beverages promotes *Candida* spp. polyene tolerance [109].

The bioactivity of *Lactobacillus brevis* AM7 during fermentation with bread hydrolysate was evaluated against the fungus (20% to 70%). Using Liquid Chromatography, nine antifungal compounds (with 10–17 amino acid residues and masses spanning 1083.6 to 1980.7 Da) were investigated, all of which were expressed in wheat protein sequences. Bread hydrolysate fermented by *L. brevis* AM7, non-fermented bread hydrolysate, and a slurry composed of water and bread combination were all used to make bread and compared with conventional wheat bread. Compared to the other pieces of bread, those fermenting hydrolysate (18 and 22% of the dough weight) had the maximum mold-free shelf life, extending up to 10 days until mold appeared. Moreover, the fermentation hydrolysate had the fewest adverse influences on bread texture, demonstrating biotechnology’s beneficial impact and potential [22]. The essential oil of *Thymus algeriensis* was studied as a possible soft cheese preservative. We devised a novel method for determining the essential oil’s ability to preserve soft cheese. During 30 days of storage at 4 °C with 25 L of essential oil introduced, there was no contamination of *Penicillium aurantiogriseum*. Minimum inhibitory concentrations for antifungals varied from 0.01 to 0.04 mg/mL range. According to the data, the oil was active with a half-maximal inhibitory activity of 0.132 mg/mL. The volatile components in the oil were determined by using gas chromatography, gas chromatography-mass spectrometry, and nuclear magnetic resonance spectrometry. The most frequent constituent in the oil was discovered to be carvacrol, which made up 80.9% of the overall amount, followed by p-cymene (7.7%) [110].

Both people and the environment are put at risk by chemical preservatives and fungicides. Bio-preservatives, such as lactic acid bacteria (LAB), on the other hand, are efficient, secure, and biodegradable, as well as add adequate beneficial health effects. The antifungal activity of strain RM1 was the highest amongst 23 rod-shaped LAB isolates collected from Egyptian traditionally fermented milk (Rayeb). Strain RM1 was distinguished from genetically similar Lactobacillus species by 16S rRNA phylogenetic analysis and distinctive phenotypic traits, indicating that it is a distinct species whereby the name *Lactobacillus* sp. RM1 is suggested. *Lactobacillus* sp. RM1 cell-free supernatant (CFS) has considerable and broad antifungal effects, mostly against toxigenic fungi and pathogenic bacteria. 

*Lactobacillus* spp. RM1 has antifungal capabilities and the ability to prolong the shelf life of wheat grains, implying that it could be used as a natural food preservative [65]. Antimicrobial substances generated or expelled by LAB can counteract foodborne illnesses, making it a possible alternative to artificial preservatives [111]. Natural preservatives such as LAB are effective, safe, and biodegradable, with added health advantages. LAB is also frequently used as a bio-preservative to increase the shelf life of food products while in storage [112,113]. Organic acids, short-chain fatty acids, hydrogen peroxide, reuterin, diacetyl, bacteriocins, and bacteriocin-like inhibitory compounds are some of the antifungal substances produced by LAB [19,65,114], *Lactococcus lactis* spp. *lactis* ATCC 19435 inhibits fungal growth and ochratoxin A synthesis in fungal growth conditions [115,116]. Antifungal compounds found in LAB have been proven effective in decreasing yeasts and molds that degrade food [117]. Therefore, to eliminate toxic fungus and increase the quality, safety, and shelf life of food and agricultural products, it is critical to look for natural, food-grade antifungal chemicals from LAB.

## 6. Major Challenges and Future Prospects

During the recent decade, tremendous progress has been made in the field of antifungal bio-preservatives. Certain constraints and knowledge gaps, however, must still be addressed. It is also worth noting that commercial cultures are scarce, presumably because the antifungal activity of any given strain is influenced by a variety of physical and chemical factors, the food manufacturing methods, and the strains’ ability to generate chemicals in situ in the food product. Health impacts and other safety problems are also key considerations that have yet to be explored for all antifungal strains. For example, safety studies should be included as a routine practice when ascribing an antifungal strain. Such analyses should provide an examination of procured resistance to antibiotics and possible biogenic amine production in compliance with safety considerations. Even though sensory and safety evaluations for antifungal strains are commonly incomplete, trying to highlight the need for further substantiation to protect the safety of using such substances in food matrices, the antifungal bio-additives mentioned are now perfectly suited to a wide range of environments, as demonstrated by their diversified in vivo and in vitro food packaging applications [118,119,120].

Creating new ready-to-use antifungal combinations, such as Gerez et al. [121] antifungal slurry, would significantly benefit food manufacturers and provide an alternate method for addressing consumer expectations. In addition, extraction and refinement methods must be standardized and quick, sensitive, repeatable, and cost-effective techniques created. In addition, the development of sensitive and quick isolation processes could lead to the discovery of new antifungal chemicals in the future. Transcriptomic methods may become a viable strategy for determining the molecular targets of antifungal compounds generated from bacteria as more genome sequences become accessible. The effects of diverse antifungal drugs should be determined using microarray or other ‘omics’ technologies, as most of these targets are unknown. Future research should improve our understanding of the genetic mechanisms and metabolic pathways of antifungal synthesis [122].

## 7. Conclusions

An alternative to chemical preservation was highlighted due to rising consumer demands of less processed and more natural foodstuffs while maintaining quality, hygiene, and shelf life. In this perspective, bacteria and fungus and their by-products are natural bioprotective agents that might be used in food to combat fungal deterioration and respond to consumer preferences and legislation. In terms of applicability, the disparity between the series of studies and the number of available microbial cultures shows that more work is needed to make their use in food products easier. Among the most important features is in situ research using tailored fungal targets for antifungal effectiveness testing and confirmation. Prior to sale, the bioprotective microorganisms’ safety, sensory properties’ neutrality, and activity constancy must all be assessed. While antifungal medications have been extensively investigated and generally demonstrated to operate cooperatively, there is still a dearth of understanding about the entire picture of which components are implicated and how they work.

## Figures and Tables

**Table 1:** Major organic acids as an antifungal agent and targeted fungal strain.

Source of Organic Acid	Organic Acid	Microbial Target	Primary Food Products	References
*Lactobacillus brevis* AM7	Lactic acid, Acetic acid	*Penicillium roqueforti* P1, *Eurotium herbariorum* CBS 117336 and *Penicillium* *albocoremium* CBS 109582	Bread	[22]
*Lactobacillus paracasei* subsp. *paracasei* SM20, *Propionibacterium jensenii* SM11	Propionic acid, 2-pyrrolidone-5-carboxylic acid, Acetic acid, Lactic acid, 3-phenyllactic acid, Hydroxyphenyllactic acid, Succinic acid	Spoilage yeasts and molds	Yoghurt and cheese surfaces	[28]
*Pseudomonas fluorescens* ZX	Butyric acid, Acetic acid, Isobutyric acid, 2-Methylbutyric acid, 3-Methylbutyric acid	*Penicillium italicum*	Citrus fruits	[29]
Seeds of *Cuminum cyminum* L.	Cuminic acid	*Fusarium oxysporum* f. sp. *niveum*	Horticultural crops, watermelon	[30]
Acetylation reaction of CH_3_COOH with H_2_O_2_	Peracetic acid	*Aspergillus flavus*, *Penicillium verrucosum*	Maize and barley grain	[31]
*Bacillus cereus*, *B. subtilis* *B. mojavensis*, *B. velezensis*	Indoleacetic acid	*Fusarium equiseti*	*Vicia faba*	[32]
*Lactobacillus plantarum IS10*, *L. brevis NCDC 02*, *L. paracasei M3*	5-Oxopyrrolidine-2-carboxylic acid, 3-(4-Hydroxyphenyl) propanoic acid, 3-Phenylpropanoic acid, Hydroxyphenyllactic acid, Dodecanoic acid	*Mucor*, *Penicillium*	Yoghurt, cheese, sour cream	[33]
*Lactobacillus fermentum* YML014	Lactic acid, Acetic acid	*Aspergillus niger*, *A. flavus*, *Candida albicans*, *Penicillium expansum*, *Zygosaccharomyces rouxii*	Fermented vegetables	[34]
*Penicillium chrysogenum* CECT 20922	Hexanoic acid Octanoic acid	*Cladosporium cladosporioides*, *C. herbarum*, *C. oxysporum*	Meat, dry-cured fermented sausages	[23,35]

**Table 2:** Phenyllactic acid-producing source and its targeted fungal strain.

Source of PLA	Microbial Target	Primary Food Products	References
*Latobacillus crustorum*	Broad range of bacterial and fungal species	Naturally fermented Chinese vegetable	[37]
*Lactobacillus brevis*, *L. plantarum*, *L. sakei*, *Leuconostoc lactis*, *Leuconostoc mesenteroide*, *Pediococcus pentosaceus*,	*Aspergillus*, *Penicillin* spp., *Candida*, *Rhodotorula* spp.	Kimchi (a fermented vegetable food product in Korea)	[41]
*Lactobacillus plantarum* KP3 *L. plantarum* KP4	Gram-positive/negative bacteria and some fungal species	*Porphyra* residues	[42]
*Lactobacillus* sp. MX3.2	*Aspergillus niger*, A. flavus, *A. oryzae*, *E. coli*, *Salmonella enterica*, *Shigela flexneri*	Mango and chilli	[43]
*Lactobacillus *plantarum argentoratensis**, *Enterococcus faecium*	Aflatoxigenic fungi	Agricultural commodities	[44]
*Lactobacillus buchneri* GBS3	Aspergillus, Penicillium, Fusarium species	Traditional Chinese pickles	[38]
*Lactobacillus plantarum* dy-1	Broad spectrum of fungi	Fermented barley extracts	[45]
*Lactobacillus *kefiri** M4, *Pediococcus *acidilactici** MRS-7	*Penicillium *expansum**	Fruits	[46]
*Lactobacillus plantarum*	*Fusarium oxysporum*, *F. fujikuroi*	Crops	[47]
*Lactobacillus brevis*, *L. plantarum*	*Phytophthora infestans*	Fermented green olives	[48]
*Lactobacillus* brevis, *L. plantarum*, *L. pentosus*	Fungi (*Candida pelliculosa* and *Penicillium digitatum*) Molds (*Penicillium* sp., *Aspergillus niger*, *Rhizopus* sp., *Fusarium oxysporum*), Yeasts (*Candida pelliculosa* and *Rhodotorula* sp.),	Fermented green olives	[49]
*Lacticaseibacillus* spp. and *Lactiplantibacillus* spp.	*Debaryomyces *hansenii**, *Torulaspora delbrueckii*, *Meyerozyma guilliermondii*	Cottage cheese	[50]
*Lactobacillus reuteri* R29	*Fusarium culmorum*	Bread system	[51]
*Pediococcus acidilactici* CRL 1753	*Aspergillus niger* CH2, *Candida tropicalis* CH6, *Penicillium roqueforti* CH4, *Metschnikowia pulcherrima* CH7	Bread	[52]
*Lactobacillus plantarum* TR7, *L. plantarum* TR71	*Penicillium expansum*, *Aspergillus flavus*	Tomato	[53]
*Lactobacillus plantarum*	*Aspergillus fumigatus*, *Penicillium roqueforti*	Chinese pickles	[54]
*Lactobacillus hammesii*	*Aspergillus *niger**, *Penicillium roqueforti*	Wheat bread	[55]
*Lactobacillus plantarum* CRL 778	*Aspergillus niger*	Fermented foods	[56]
*Lactobacillus *fermentum**, *L. plantarum*	*Aspergillus* and *Penicillium* genera	Fermented and dried cocoa beans	[57]

**Table 3:** Cyclic dipeptides identified as antifungal agents.

Sources of CDP	Identified CDP	Microbial Target	References
*Lactococcus lactis* subsp. *cremoris*	cyclo(Leu-Pro)	Fungal Species	[77]
*Aciculosporium take*	cyclo(L-pro-L-Phe), cyclo L-pro-L-Leu), cyclo(L-pro-L-Ile)	Fungal species	[78]
*Epicoccum nigrum* M13	cyclo(L-Pro-L-Ile), cyclo(L-Pro-L-Val), cyclo(L-Pro-L-Tyr), cyclo(L-Pro-L-Phe),	Fungal and bacterial species	[79]
*Bacillus amyloliquefaciens* Q-426	cyclo(L-Pro-L-Phe), cyclo(L-Pro-D-Phe), cyclo(D-Pro-D-Phe), cyclo(D-Phe-L-Pro)	Broad range of fungi	[80]
*Bacillus amyloliquefaciens* subsp. *plantarum* strain FZB42	*cis*-cyclo(L-Pro-L-Ile), *cis*-cyclo(L-Pro-L-Leu), *cis*-cyclo(L-Pro-L-Phe), *cis*-cyclo(L-Pro-L-Pro), *cis*-cyclo(L-Pro-L-Val),	Filamentous fungi	[74]
*Bacillus velezensis* AR1	5-*N*-tyrosinylornithine	*Monilinia fructicola* and *Colletotricum goeosporioides*	[73]
Prenylation of tryptophan with cyclic dipeptides at C7 position by 7-Dimethylallyl	cyclo(L-Trp-Gly), cyclo(L-Trp-L-Ala), cyclo(L-Trp-L-Phe), cyclo(L-Trp-L-Leu), cyclo(L-Trp-L-Pro), cyclo(L-Trp-L-Trp), cyclo(L-Trp-L-Tyr)	*Aspergillus flavus*, *Candida albicans*, *Fusarium oxysporum*, *Alternaria brassicae*, *Rhizoctonia solani*, *Penicillium expansum*	[81]
*Pediococcus pentosaceus*	Hexahydro-7-hydroxy-phenylmethyl	*Aspergillus niger*	[82]
*Lactobacillus rhamnosus*	9-amino acid peptide (a derivative of α_s2_-casein)	*Mucor racemosus*, *Rhodotorula mucilaginosa*	[60]
*Paenibacillus* sp. MS2379	Fusaricidins along with amino acid residues of γ-aminobutyric acid and serine	Broad array of fungal pathogens	[83]
*Lactobacillus plantarum* LBPK10	cyclo(Val-Pro), cyclo(Tyr-Pro), cyclo(Ser-Pro),	Broad range of fungal species	[84]
*Lactobacillus plantarum* LBPK10	*cis*-cyclo(L-Leu-L-Hyp), cyclo(Phe-Pro), cyclo(Leu-Pro)	Bacterial, virus and fungal pathogen.	[84]
*Lactobacillus casei* AST18, *L. plantarum* AF1, *L. Plantarum* MiLAB 393,	2,6-diketopiperazines and their derivatives, 2,5-diketopiperazines, 2,3-diketopiperazines	Fungi and Gram-positive/negative bacteria	[85]
*Pediococcus pentosaceus*	Non-pediocin-like peptides	*Fusarium graminearum*	[76]
*Bacillus cereus* subsp. *thuringiensis*	cyclo(D-Pro-L-Met) cyclo(D-Pro-D-Tyr)	*Rhizoctonia solani*, *Fusarium oxysporum*, *Penicillium expansum*	[71]
*Lactobacillus plantarum*	cyclo(Tyr-Pro), *cis*-cyclo(L-Leu-L-Pro), *cis*-cyclo(L-Val-L-Pro), *cis*-cyclo(L-Phe-L-Pro)	Broad spectrum of fungi, bacteria, and virus	[72]
*Bacillus* spp.	cyclo(L-Pro-L-Tyr), cyclo(D-Pro-L-Leu), cyclo(L-Pro-L-Met), cyclo(D-Pro-L-Phe), cyclo(L-Pro-D-Tyr), cyclo(L-Pro-L-Phe)	*Rhizoctonia solani*, Aspergillus flavus, *Candidaalbicans, *Penicillium expansum**, *Fusarium oxysporum*	[86]
